# ERRATUM

**DOI:** 10.5935/0103-507X.20160018

**Published:** 2016

In the article **Influence of different degrees of head elevation on respiratory mechanics in mechanically ventilated patients**, DOI number: 10.5935/0103-507X.20150059, published in **Revista Brasileira de Terapia Intensiva** 2015;27(4):347-52, page 347 “Vanessa Silva Salgado” should be read as “Vanessa Salgado Silva”.

In the article **Delirium in intensive care unit patients under noninvasive ventilation: a multinational survey**, DOI number: 10.5935/0103-507X.20150061, published in **Revista Brasileira de Terapia Intensiva** 2015;27(4):360-8, pages 365 and 366, Discussion, 10th paragraph, lines 10 and 25, “acute renal failure” should be read as “acute respiratory failure”.

